# Genitalic Differentiations in *Neoleucinodes elegantalis* (Gueneé) (Lepidoptera: Crambidae) Associated with *Solanaceae* Crops in Ecuador

**DOI:** 10.3390/insects8030091

**Published:** 2017-08-31

**Authors:** Michelle Noboa, William Viera, Ana Díaz, Wilson Vásquez, Lenin Ron

**Affiliations:** 1 Instituto Nacional de Investigaciones Agropecuarias (INIAP), Programa Nacional de Fruticultura, Tumbaco, Quito 170184, Ecuador; noboamichelle@gmail.com (M.N.); leninron@agro.uba.ar (L.R.); 2 Corporación Colombiana de Investigación Agropecuaria (CORPOICA), Centro de Investigación Obonuco, Km 5, Vía Pasto, Pasto 520038, Colombia; anaelizabethd@gmail.com; 3 Facultad de Ingeniería y Ciencias Agropeuarias, Universidad de las Américas (UDLA), José Queri, Quito 170137, Ecuador; wilovasquez@yahoo.com; 4 Facultad de Medicina Veterinaria y Zootecnia, Centro Internacional de Zoonosis. Universidad Central del Ecuador, Quito 170521, Ecuador

**Keywords:** genitalia, morphometry, moth, Solanaceae, subspecies

## Abstract

*Neoleucinodes elegantalis* (Guenée) is an oligophagous species of plants in the Solanaceae family that has a broad geographical distribution in the tropical zones of South America. It is the most important insect pest of naranjilla (*Solanum quitoense* Lamarck), a crop grown in threatened areas of the tropical old-growth forest in Ecuador. In this study, two host-specific populations of *N. elegantalis* were collected from infested fruit of naranjilla and tree tomato (*Solanum betaceum* Cavanilles) in different locations. Sexually virgin adult insects (93 females and 103 males) were dissected to extract their genitalia to measure 12 morphological variables in females and six in males, resulting in six and four informative variables respectively. Using univariate and multivariate analysis of variance, it was found that the Solanaceous host was the main factor differentiating the area measurements of the seventh abdominal segment and ostium bursae in female genitalia, and cornuti length in male genitalia. Principal components generated with these measurements were employed in a logistic regression model for the classification of the Solanaceous host. Female genitalia of individuals from *S. betaceum* showed significantly larger ostium bursae relative to female genitalia from *S. quitoense*. For males, individuals collected from *S. betaceum* showed longer cornuti length than samples collected from *S. quitoense*. The results suggest genotypic differentiation according to the Solanaceous host or phenotypic plasticity in *N. elegantalis*. Further molecular and bio-geographical studies are needed to properly differentiate *N. elegantalis* populations that would help in the control of this pest.

## 1. Introduction

Recent studies on the biology of the fruit borer *Neoleucinodes elegantalis* (Guenée, 1984) (Lepidoptera: Crambidae) carried out in Latin America, have found that this pest is an oligophagous species of plants in the Solanaceae family, and is distributed in almost all countries of South America infesting various crops of economic importance, such as *S. lycopersicum* (tomato), *S. betaceum* (tree tomato), *S. capsicum* (pepper), and *S. quitoense* (naranjilla) [1,2,3,4].

**Figure 1:**
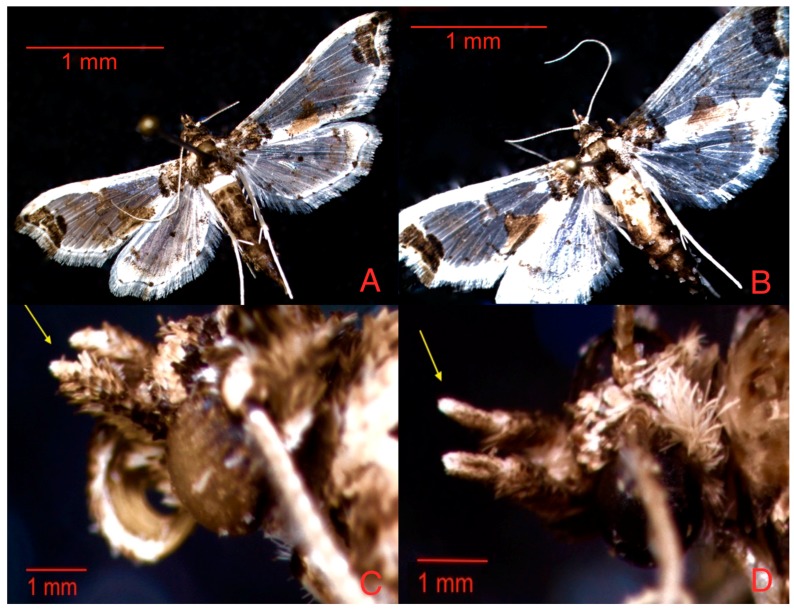
Adults of *N. elegantalis*; (**A**) male; (**B**) female; (**C**) labial palpus of male adult; and (**D**) labial palpus of female adult.

According to Capps (1948), this species has the following characters: a simple antenna; slightly annulate; labial palpus upturned; remarkably shorter maxillary palpus in males than in females; frons evenly rounded; a posterodorsal area of the head with white scales predominant; a dark fuscous brown thorax (dorsal view) that is white in ventral view; an abdomen with a conspicuous white band composed of all the first and variable portions of the second a third segments; white hyalinate wings with conspicuous squamous areas of cinnamon brown [5], as shown in Figure 1.

In Ecuador, this pest is present in all the regions where naranjilla (*S. quitoense*) is grown. The fruit borer directly affects the fruit pulp by boring and feeding on the fruit mesocarp, producing galleries inside the developing berries [4] resulting in significant losses in production and fruit quality.

*N. elegantalis* is endemic in tropical rainforest regions of Ecuador, specifically in the Andean foothills where fruit of native Solanaceae species such as naranjilla and tree tomato can be found. In fact, nearly 100% of naranjilla is grown in these areas. Therefore, farmers are obligated to apply insecticides on the fruit, and in many cases to look for new places in old-growth forest to relocate their orchards. As a result, serious damage to this ecosystem is created because of agricultural activities.

There is evidence that *N. elegantalis* has a dichotomous behavior with respect to the preference of host species; it can choose one or the other but not both [6]. Studies in Colombia have identified four biotypes of *N. elegantalis*, correlated with different Solanaceae hosts. Those biotypes were separated by the localization in the Eastern and the Western Colombian Andes [6]. Consequently, we hypothesize that different biotypes of *N. elegantalis* exist in Ecuador due to host specificity in different ecological areas, due to sympatric and/or allopatric separation of this species. Thus, the present study aims to evaluate the possibility of different biotypes of *N. elegantalis* in Ecuador, using genitalia morphometric measures of virgin adult specimens of *N. elegantalis* found in two Solanaceae fruit crops, *S. quitoense* and *S. betaceum*, collected from different areas of Ecuador.

## 2. Material and Methods

### 2.1. Sample Collection and Preparation

Infested fruits of *S. quitoense* from commercial orchards and *S. betaceum* from abandoned orchards that were close to *S. quitoense* orchards were collected with larvae inside and were sent to the Entomology laboratory in INIAP-Tumbaco. We analyzed 196 *N. elegantalis* (103 males, 93 females) collected from different *S. quitoense* and *S. betaceum* crop areas located in the Eastern and Western foothills of the Ecuadorian Andean rainforest and were analyzed to identify potential morphological intraspecific differences. Genitalia were used because such characters are much more conserved than external characters, and because they are insignificantly influenced by environmental factors [7,8].

Infected fruits were placed in growth chambers with controlled environmental conditions (temperature 22 ± 1 °C, 55% relative humidity and a photoperiod of 12 h of light and 12 h of darkness) to complete their larval, pupal and adult development.

*N. elegantalis* were collected from eight localities in five provinces of Ecuador (Table 1). Both *S. quitoense* and *S. betaceum* were found together only in Los Bancos, Pichincha, and in El Chaco, Napo; in all other localities, *N. elegantalis* was found only on *S. quitoense* (Table 1).

### 2.2. Slide Preparation and Measurement

The adult moths were sacrificed in a hermetic container with ethyl acetate gas for about 60 s. Later, genitalia were prepared using the protocol described by Hardwick in 1950 [9]. Genitalia slides were photographed using an *Infinity 1* digital camera attached to an Olympus SZ stereomicroscope; the measures of the variables were recorded using the software infinity analyze, release 6.2.0.

**Figure 2:**
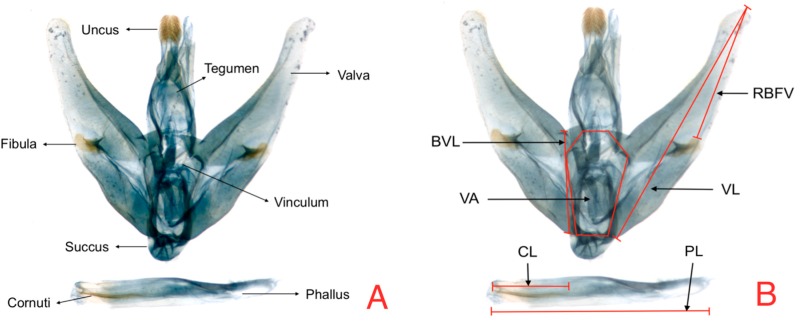
Male genitalia (**A**) Anatomic structures (**B**) Measures: PL = phallus length CL = cornuti length; VL = valva length; VA = Vinculum area; RBFV = ratio of the fibula position in the valve; BVL = basal valva length.

In relation to male genitalia, the characters measured were the phallus length (PL), cornuti length (CL), valva length (VL), valva width (VW), and vinculum area (VA), plus the fibula–valva apex length (Figure 2). With the original measures of the structures of the genitalia, three descriptive variables were formed. The variable (RLAV) expresses the proportion of the length of the valva relative to its basal width, using the VL valva length/BVL basal valva length relationship. Fibula position in the valva (FPV) expresses the ratio of distance from the fibula to total valva length, using the RBFV/VL relationship. Ratio cornuti length (RCL) based on a relation with phallus length (PL) was obtained by determining the PL/CL relationship. Finally, vinculum area (VA) was determined by calculating the hexagonal area.

**Figure 3:**
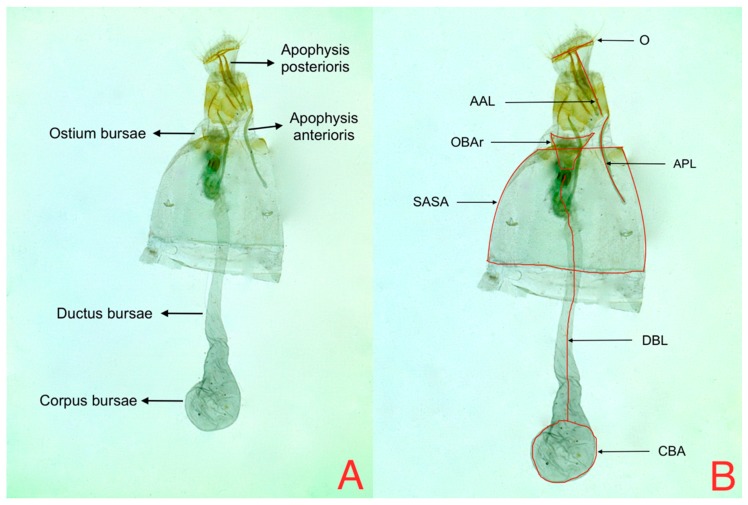
Female genitalia (**A**) Anatomic structures and (**B**) Measures: AAL = apophysis anterioris length; APL = apophysis posterioris length; SASA = seventh abdominal segment area; DBL = ductus bursae length; CBA = corpus bursae area; OBAr = ostium bursae area.

With respect to female genitalia, all variables were measured following the method proposed by Diaz et al. 2015 [6]. Ductus bursae length (DBL) was measured from the constriction of the ostium bursae to the beginning of the corpus bursae; apophysis posterioris length (APL) was estimated as the relation between apophysis posterioris and the right anterior wing length (LRAW) where RAPL = APL/LRAW; apophysis anterioris length (RAAL) was measured as the relation between apophysis anterioris (AAL) and the right anterior wing length (RWL) where RAAL = AAL/RWL. Ostium bursae area (OBAr) was calculated using the isosceles triangle area formula; the seventh abdominal segment area (SASA) was determined by calculating the trapezium area and finally the corpus bursae (CBA) area was determined by calculating the elliptical area (Figure 3). The areas of the last three variables were automatically calculated by the software using the formulas described above.

### 2.3. Statistical Analysis

Data analyses were performed using univariate ANOVA and multivariate MANOVA statistical analyses. Outlier detection was performed using Mahalanobis distance. Barlett’s test was applied for each variable to examine variance homogeneity. Likewise, ANOVA and MANOVA analysis was performed to find significant differences among host and zones considering the full set of variables for females and males. F-test from Pialli’s trace statistics was chosen to evaluate individual significant factors from the MANOVA. Classification of the specimens was done using Principal component logistic regression, using eight variables for females and five variables for males [10]. The size effect of orthogonal linear compose variables (component dimensions) was tested for each Solanaceous host (*S. betaceum* or *S. quitoense*) usin FactoMiner package [11]. The statistical significance of each regression parameter was set at *p* < 0.05. All analyses were done using *R* software 3.3.1 (https://www.r-project.org).

## 3. Results

### 3.1. Analysis of Variance for Solanaceae Plant and Zone

**Figure 4:**
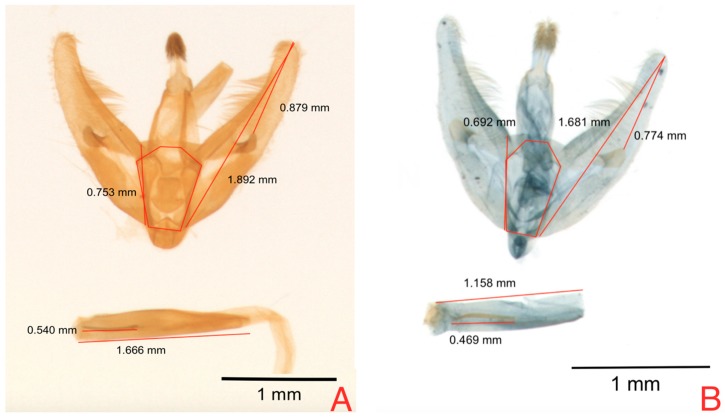
Morphometric differences in male genitalia of *N. elegantalis* from two Solanaceous hosts; (**A**) naranjilla (*S. quitoense*); and (**B**) tree tomato (*S. betaceum*).

For male genitalia, significant differences were found for host plant factor in the cornuti length (RCL) in the ANOVA analysis (Table 2). Additionally, there were differences for the zone factor in the variable RCL where specimens collected in Eastern Andes lands presented a more reduced length for this variable (Figure 4).

MANOVA analysis of males of *N. elegantalis* found highly significant differences between host plant and zone variables, (Table 3). These findings are consistent with the study by Díaz et al. 2015 in Colombia [6], where the source of food was the main factor that discriminated the morphological differentiation among the female population of *N. elegantalis*, giving support to sympatric differentiation. In this study, zone was also found as an important factor for male differentiation. In both cases, sympatric differentiation seems to be the main force in the genetic dynamics of this species, as the source of food seems to determinate the size of the morphological structures. Food source represented the highest source of variation among the populations studied and differentiated four population groups of *N. elegantalis* in Ecuador.

**Figure 5:**
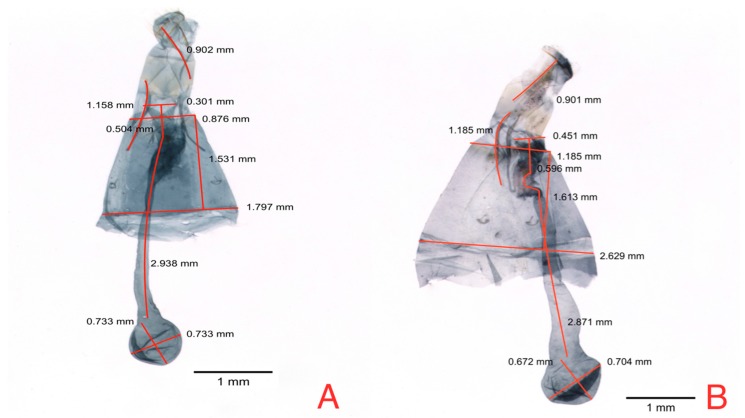
Morphometric differences in female genitalia of *N. elegantalis* from two Solanaceous hosts; (**A**) naranjilla (*S. quitoense*) and (**B**) tree tomato (*S. betaceum*).

ANOVA analysis of female genitalia (Table 4) variables found statistical differences (*p* < 0.05) for Solanaceous host for two out of six evaluated variables, OBAr and SASA (Figure 5). These results are consistent with the results obtained by Díaz et al. 2015, which mention that SASA shows the highest variability, suggesting that this variable might be useful as a morphological marker to differentiate from which host the individuals of this species come [5,12].

On the other hand, the CBA variable showed statistical differences (*p* < 0.05) for the interaction between host and zone effect. For the zone, there were no mean differences, which suggests that climatological similarities between zones do not cause differentiation among individuals.

MANOVA analysis found significant differences only for the Solanaceous host between female populations from *S. betaceum* and *S. quitoense* (Table 5), suggesting that sympatric differentiation may take place in contrast to allopatric differentiation. A possible explanation for this nonstructural geographical differentiation of *N. elegantalis* might be the relative facility and connectivity between Eastern and Western sides of the Andes in Ecuador. According to Díaz et al. 2013 [1], in Colombia, there were geographic differentiations of *N. elegantalis* likely due to deeper isolation of different biotypes in comparison with the mobility among different areas that exist in Ecuador.

### 3.2. Principal Component Host Classification

Principal component logistic regression for male genitalia of *N. elegantalis* used in the classification of specimens coming from the two Solanaceae hosts (Table 6), resulted in statistical significance for PC3.

**Figure 6:**
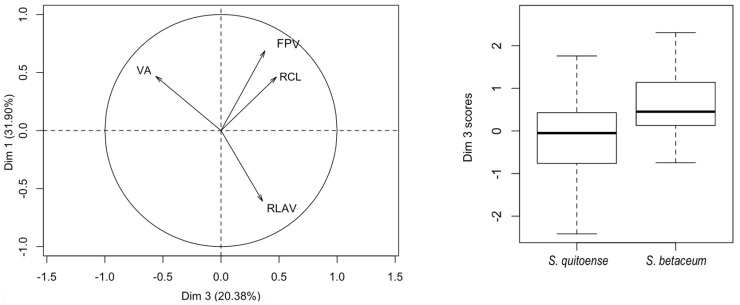
Biplot and boxplot of PC3 and PC1 components in the classification of *N. elegantalis* males hosted in two Solanaceae families (*S. quitoense* and *S. betaceum*).

Table 7 presents the loadings of this component, which shows the contrast between VA and RBFV. Figure 6 represents the dispersion of measures for this component of the individuals in both hosts and clearly shows that individuals from tree tomato have larger size than individuals from naranjilla.

**Figure 7:**
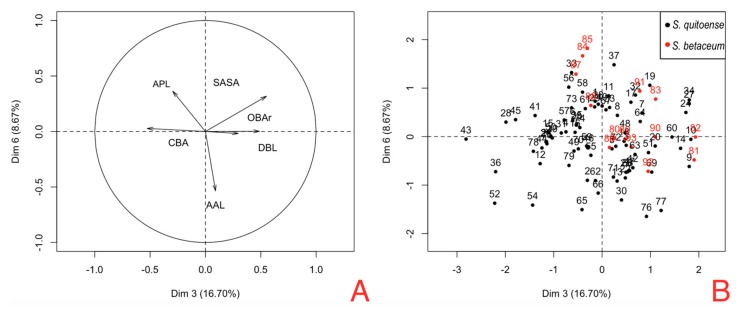
Principal component analysis; (**A**) Biplot of PC3 and PC6; (**B**) PCA plot components in the classification of *N. elegantalis* females hosted in two Solanaceae families (*S. quitoense* and *S. betaceum*).

The principal component logistic regression for female genitalia of *N. elegantalis*, in the two Solanaceous hosts (Table 8), identified two significant components, PC 3 and PC 6, with the results of each variable shown in Figure 7A. PC3 shows the contrast between CBA vs. OBAr, and DBL vs. SASA (Table 9). PC6 shows the contrast of RAAL between females on naranjilla with bigger RAAL in comparison to females on tree tomato. Figure 7B shows the position of females hosted in tree tomato (red dots) and females hosted in naranjilla (black dots); these components show that tree tomato females have a bigger SASA and OBAr and smaller CBA and RAPL. Figure 5 shows that the areas occupied by OBAr and SASA are bigger than other structures, showing that females on tree tomato have a larger capacity in the abdominal region.

## 4. Discussion 

Most researchers identify species using operational methods, mainly based on phenotypic morphological evidence or on molecular phylogenetics [13,14]. This has generated a tendency for taxonomists to divide allopatric populations of the species into distinct species, based on minor morphological or karyotypic differences [8,13]. The genetic divergence and evolution of new species within the geographic range of a single population contrasts with the well-established doctrine that speciation occurs when populations become geographically isolated [15]. Although there is important theoretical support for sympatric speciation [16], this mode of diversification remains controversial, at least in part because there are few well-supported examples [17]. In those cases, evidence of changes in the morphometry of genitalia in both sexes of *N. elegantalis* was found according to the host, given that the reproduction in each one of both biotypes may be facilitated by partner discrimination and the avoidance of mating interference. Similar findings analyzing genitalia have been suggested in other species such as snails, in northern Okinawa Island, Japan [18].

Evidence of sympatric speciation of *N. elegantalis* using morphological characters related to host specificity was also found in this study. This information will help to understand the movement of this insect between fields, orchards and regions of Ecuador. Furthermore, our results can be used to distinguish invasive populations from Colombia, as all variables, except in ArOB Ecuadorian individuals, show smaller structures than Colombian specimens, taking the study of Díaz and collaborators in 2015 as a reference [6]. The results of this study will contribute to control strategies such as the use of pheromones according to the ethological behavior of this moth. In the future, molecular studies could be carried out to determine if morphometric differences are caused by genetic divergence or phenotypic plasticity because individuals belonging to the same species (genotype) can show different morphometric structures depending on environmental conditions.

## 5. Conclusions

In this study, the Solanaceous host was found to be a factor that may discriminate populations of *N. elegantalis* based on genital morphological differentiation. Among the Solanaceae fruit crops, *S. quitoense* is the main host for this species, however *S. betaceum* might be considered as an alternate host in Ecuador. The size of the genital structures represented the major source of variation among the studied populations infecting *S. quitoense* and *S. betaceum*; this made it possible to identify two population groups (biotypes) of *N. elegantalis*, both in males and females. This discrimination of biotypes is important for the control strategies that can be applied to moths of *N. elegantalis* in critical places such as the Andean foothills.

## Figures and Tables

**Table 1:** Sampling areas of the study in Ecuador, South America.

Locality	Province	Zone	Altitude (Masl)	Host Plant	Number of Specimens	
					Female	Male
Los Bancos	Pichincha	Western Andes	681	*S. quitoense*	11	10
				*S. betaceum*	8	16
Rio Negro	Tungurahua	Eastern Andes	1242	*S. quitoense*	4	6
El Chaco	Napo	Eastern Andes	1600	*S. quitoense*	8	7
				*S. betaceum*	6	6
Awayaku			735	*S. quitoense*	9	11
Río verde	Carchi	Western Andes	814	*S. quitoense*	8	9
Palora	Morona Santiago	Eastern Andes	1358	*S. quitoense*	9	9
Guagua Sumaco	Napo		1053	*S. quitoense*	13	11
Cumandá	Napo		1123	*S. quitoense*	9	11

**Table 2:** ANOVA test for the descriptive variables of the male genitalia of *N. elegantalis* collected in two hosts (*S. quitoense* and *S. betaceum*) and two zones (Western Andes and Eastern Andes).

		Variables							
		VA		RLAV		FPV		RCL	
Source of variation		F. calc.	Pr (>F)	F. calc.	Pr (>F)	F. calc.	Pr (>F)	F. calc.	Pr (>F)
Zone	1	1.26	0.26	1.51	0.15	1.33	0.22	2.07	0.04 **
Host plant	1	2.41	0.12	0.28	0.59	1.02	0.31	16.67	0.00 **
Host plant × zone	1	0.05	0.82	2.25	0.13	0.33	0.56	1.58	0.21
Error	98								
Total	101								
Average (mm)		2.11		0.64		2.07		2.96	
C.V. (%)		22.16		3.82		6.8		13.53	

Note: F. calc. = F calculated value; Pr (>F) = *p* value; C.V. = coefficient of variation; ** = high statistical significance; VA = Vinculum area; RLAV = relation between valva length and basal valva length; FPV = ratio of the fibula position in the valve; RCL = relation between phallus length and cornuti length.

**Table 3:** Decision criteria for the multivariate variance analysis (MANOVA) in the metrics of male genitalia of *N. elegantalis*.

Source of Variation	GL	Pillai Criteria	Approx F	Pr (>F)
Host plant	1	0.1763	5.137	0.0008 **
Zone	1	0.0983	2.6188	0.0396 *
Host plant × zone	1	0.0194	0.4771	0.7524
Error	99			

Note: GL = freedom degrees; Pr = value *p* greater than F; * statistical difference; ** = high statistical significance.

**Table 4:** ANOVA test for the descriptive variables of the female genitalia of *N. elegantalis* collected in two hosts (*S. quitoense* and tree *S. betaceum*) and two zones (Western Andes and Eastern Andes).

		Variables											
		DBL		RAPL		RAAL		OBAr		SASA		CBA	
Source of variation	Df	F. calc.	Pr (>F)	F. calc.	Pr (>F)	F. calc.	Pr (>F)	F. calc.	Pr (>F)	F. calc.	Pr (>F)	F. calc.	Pr (>F)
Zone	1	0.28	0.597	3.846	0.053	3.737	0.056	0.03	0.859	1.057	0.306	1.076	0.302
Host plant	1	0.048	0.826	0.002	0.957	0.396	0.53	8.12	0.005 **	4.581	0.035 *	1.722	0.193
Host plant × zone	1	1.728	0.192	0.89	0.348	0.044	0.833	0.014	0.904	1.112	0.294	4.487	0.037 *
Error	88												
Total	92												
Average		2.284		0.083		0.115		0.087		2.171		0.237	
C.V. (%)		19.438		12.885		15.141		21.561		14.711		45.060	

Note: F. calc. = F calculated value; Pr (>F) = *p* value; C.V. = coefficient of variation; * statistical difference; ** = high statistical significance; AAL = apophysis anterioris length; APL = apophysis posterioris length; SASA = seventh abdominal segment area; DBL = ductus bursae length; CBA = corpus bursae area; OBAr = ostium bursae area.

**Table 5:** Multivariate analysis of variance (MANOVA) in the metrics of female genitalia of *N. elegantalis*, collected in two hosts (*S. quitoense* and *S. betaceum*) and two Zones (Western Andes and Eastern Andes).

Source of Variation	GL	Criteria of Pillai	Approx F	Pr (>F)
Host plant	1	0.1904	2.9799	0.0114 *
Zone	1	0.1158	1.6596	0.1425
Host plant × zone	1	0.1199	1.7266	0.1262
Error	81			

Note: GL = freedom degrees; Pr (>F) = *p* value greater than F; * = statistical significance.

**Table 6:** Principal Components (PC3) and the anatomical structures of males of *N*. *elegantalis*.

Component	Estimate	Std. Error	*z* Value	*p* Value
Intercept	−1.47	0.29	−4.94	0.001 **
PC 1	0.26	0.23	1.11	0.264
PC 2	−0.03	0.28	−0.11	0.908
PC 3	−1.21	0.35	−3.45	0.005 **
PC 4	0.11	0.31	0.36	0.716

Note: Std. Error = standard error; ** = high statistical significance.

**Table 7:** Principal Components (PC3) and the anatomical structures of males of *N*. *elegantalis*.

Variable	PC 3
VA	−0.56
RCL	0.71
RLAV	0.41
FPV	0.37

Note: VA = Vinculum area; RCL = relation between phallus length and cornuti length; RLAV = relation between VL valva length and BVL basal valva length; FPV = ratio of the fibula position in the valve.

**Table 8:** Logistic regression estimates between the classifications components of *N*. *elegantalis* females–host plant and the size of the Principal Component structures.

Component	Estimate	Std. Error	*z* Value	*p* Value
Intercept	−2.34	0.45	−5.16	0.0001 **
PC 1	0.09	0.24	0.39	0.695
PC 2	−0.27	0.29	−0.92	0.352
PC 3	−0.91	0.40	−2.26	0.023 *
PC 4	0.60	0.36	1.65	0.098
PC 5	0.52	0.38	1.37	0.167
PC 6	−1.14	0.49	−2.30	0.021 *

Note: Std. Error = standard error; * statistical significance; ** = high statistical significance.

**Table 9:** Principal Components (PC3 and PC6) and the anatomical structures of females of *N*. *elegantalis*.

Variables	PC3	PC6
DBL	−0.29	0.00
RAAL	0.00	0.74
RAPL	0.29	−0.50
OBAr	−0.48	0.00
SASA	−0.50	−0.44
CBA	0.52	0.00

Note: DBL = ductus bursae length; RAAL = apophysis anterioris length; RAPL = apophysis posterioris length; OBAr = ostium bursae area; SASA = seventh abdominal segment area; CBA = corpus bursae area.
